# Mixed-Integer Linear Programming Formulation with Embedded Machine Learning Surrogates for the Design of Chemical Process Families

**DOI:** 10.1021/acs.iecr.4c03913

**Published:** 2025-04-11

**Authors:** Georgia Stinchfield, Natali Khalife, Bashar L. Ammari, Joshua C. Morgan, Miguel Zamarripa, Carl D. Laird

**Affiliations:** † Department of Chemical Engineering, Carnegie Mellon University, Pittsburgh, Pennsylvania 15213, United States; ‡ National Energy Technology Laboratory (NETL), Pittsburgh, Pennsylvania 15236, United States; § NETL Support Contractor, Pittsburgh, Pennsylvania 15236, United States

## Abstract

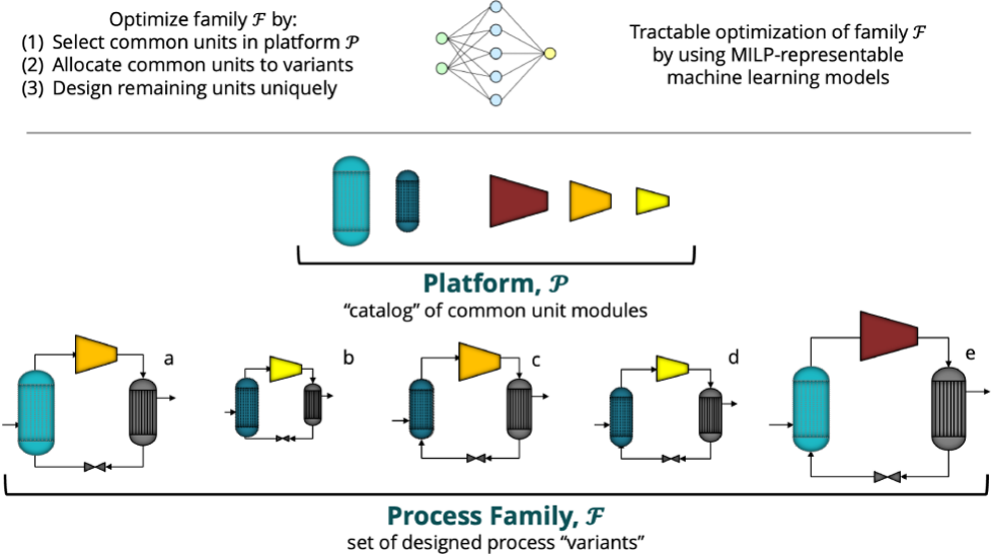

There is a need for design strategies that can support rapid and widespread deployment of new energy systems and process technologies. In a previous work, we introduced *process family design* as an alternative method to traditional and modular design approaches. In this article, we develop piecewise linear surrogates using Machine Learning (ML) models and the Optimization and Machine Learning Toolkit (OMLT) to show how process families can be designed to reduce manufacturing costs and deployment timelines. We formulate this problem as a nonlinear Generalized Disjunctive Program (GDP), which, following transformation, results in a large-scale mixed-integer nonlinear programming (MINLP) problem. This large-scale problem is intractable using traditional MINLP approaches. By using ML surrogates to predict required system costs and performance indicators, we can approximate the nonlinearities in the GDP to generate an efficient mixed-integer linear programming (MILP) formulation. We apply the ML surrogate approach to two case studies in this work. One case study involves designing a family of carbon capture systems to cover a set of different flue gas flow rates and inlet CO_2_ concentrations, while the second case study focuses on a water desalination process, where we design a family of these processes for a variety of salt concentrations and flow rates. In both of these case studies, our approach based on ML surrogates is able to find optimal solutions in reasonable computational time and yield solutions comparable to those of a previously reported approach for solving the problem.

## Introduction

As process engineers, when we consider designing multiple instances of a particular process system, the conventional approach considers each process instance to be an independent design task. However, when the number of processes that must be designed increases, this quickly becomes expensive, particularly due to the high costs associated with unique production. One cost-mitigating approach could be modular design, which introduces manufacturing standardization to significantly reduce the cost. However, while modular design provides a high level of manufacturing standardization, i.e., it targets economies of learning cost savings, it fails to fully exploit economies of scale. Rather than using a conventional or modular design approach, we introduce elements of product family design to achieve manufacturing benefits similar to those of modularity while maintaining levels of process customization.

We will show in this paper that the product (and process) family design problem can be formulated as a large-scale Mixed-Integer Nonlinear Program (MINLP) or, more specifically, a nonlinear Generalized Disjunctive Program (GDP). We demonstrate how we can effectively replace the nonlinearities with multivariate piecewise linear representations derived from ML models, yielding an MILP formulation rather than the original large-scale MINLP. Modern ML tools, such as Keras^1^ or TensorFlow,^2^ train various ML models efficiently. OMLT^3^ provides an automatic transformation of many common ML models (trained and built on a variety of these open-source ML tools) into math programs that exactly represent the trained ML model in Pyomo.^4^ These models are embedded within an overall optimization problem modeled in Pyomo^4^ as constraints or incorporated within the objective function. OMLT provides the necessary framework for seamlessly integrating ML models into the overall optimization decision framework.

In product family design, the family is “a set of products that share one or more common element(s) yet target a variety of market segments”.^5^ This manufacturing approach is actively exploited across many industries. Consider the automotive industry, for example. Modern automotive manufacturers want to offer a wide variety of products (e.g., different model cars, such as minivans, sports cars, hybrids, etc.). A single automotive manufacturer can significantly lower the cost of producing such a catalog of different products by standardizing elements that they all share.^6^ For example, a pick-up truck and a minivan (while both clearly different cars and targeted toward different customers) both require a steering wheel. Product family design might have both model cars share the same steering wheel design; it does not impact the clear differentiation between a pick-up truck and a minivan, but it does enable the manufacturer to save time and money at the manufacturing level. As process vendors tackle the broad deployment of large numbers of chemical processes, they face similar design goals and can therefore benefit from similar manufacturing approaches.

Our work has focused on integrating the concepts developed for product family design into the area of process design and manufacturing. In this scenario, the “products” are the instances of the process that we must design. Each process instance is termed a process variant, where each variant is associated with a set of conditions that the process design must meet. The common element(s) are a specified subset of the unit modules within each process variant that will share designs. For example, one of our case studies considered the design of a carbon capture system (CCS). Typically, a CCS is designed for a specific point-source capture location (i.e., coal-fired power plant, natural gas combined cycle, cement plant, refinery, etc.) mainly based on the flue gas conditions and flow rate. Process family design has the potential to accelerate the deployment of CCS for industrial processes through the simultaneous design of process families that share common elements.

Our proposed optimization formulation simultaneously determines the individual designs of the common units offered in the platform (i.e., if we want a platform with only two absorber designs, what should those designs be) and which of those common units each process variant should use (i.e., for each of 12 process variants, which of the two absorber designs should each variant use). Previously, we proposed formulating this problem as a nonlinear GDP. We approached this nonlinear GDP by reformulating it as an MILP based on full discretization of the design space.^7,8^ We have expanded on this approach, proposing another MILP reformulation of the nonlinear GDP that now avoids using discretization within the optimization formulation, instead using piecewise linear ML surrogate models that are trained on the discretization data.^9,10^ In one case study, we show significantly decreased precomputation requirements for simulations, and we can search within an approximation of the continuous design space rather than being restricted to prespecified discretized options. In this paper, we further demonstrate this piecewise linear surrogate approach to optimize a process family of carbon capture systems and water desalination systems using a combination of neural networks (NN) with Rectified Linear Unit (ReLU) activation functions, gradient-boosted decision trees, and linear model decision trees as surrogates.

## Literature Review

Typical process system design approaches follow one of two main methods: a modular approach or a conventional one-off design approach (e.g., each design is developed for a specific application and manufactured once). Modular design approaches are time-efficient and generate cost savings due to manufacturing standardization.^11^ On the other hand, conventional design yields reduced costs by optimizing the design for a particular installation.^12,13^ In general, modular design relies on economies of *learning* whereas conventional design focuses more on economies of *scale*.

Modularity exploits economies of learning by building chemical plants with a numbering-up approach. This method relies on producing a set of base units that can be produced and assembled in numbers and stacked together (i.e., numbered-up) to achieve the desired product production capacity.^11^ Economies of learning (sometimes referred to as economies of *numbers*) play a major role in cost savings associated with this approach.^14^ The phenomenon was first observed and documented by Wright,^15^ reporting how the per-unit cost of manufacturing an airplane decreased with respect to the total unit count. Economies of learning is captured mathematically by the learning curve, which is a nonlinear, monotonically decreasing relationship between the base cost of a product and the number of times that specific product has been produced.^16−18^ Gazzaneo et al.^19^ developed a techno-economic framework for costing intensified modular systems that takes into account economies of learning. In previous work, we designed a process family of carbon capture systems while considering the explicit cost savings derived from economies of learning in the objective function.^20^ Using literature values for parameters in economies of learning correlations, our results showed significant cost savings for designing and deploying a family of carbon capture systems, demonstrating the importance of this cost-saving paradigm.

Economies of scale has been a core tenet of chemical plant design for decades.^12,13^ The phenomenon can be distilled down to the idea that constructing larger chemical plants makes capital expenditure (CAPEX) more capital-cost efficient and improves total resource utilization, thus, reducing operating expenditure (OPEX). A conventional design approach aims to fully harness the benefits of economies of scale, optimizing each element of a process system to match the intended installation.

Conventional design of a process system is at odds with a modular approach. Conventional design fails to effectively capture economies of learning, but modular design fails to capture economies of scale. While Chen and Grossmann^21^ propose a Generalized Disjunctive Program (GDP) to select between a more modular design or a conventional approach, an alternative is to develop a new approach altogether that incorporates elements of both modularity and conventional design. Recently adapted for application to process system design^7^ is product family design, which has shown documented success across a variety of industrial settings.^5^

Product family design captures the benefits of manufacturing standardization, akin to modularity, since a large number of product variants share designs for their common unit module types. Product family design has proven beneficial in a variety of industries; companies such as Nissan, Toyota, and Boeing have reported substantial cost and time savings by using this approach.^5^ The car companies Nissan and Renault developed a financially beneficial relationship for car parts production based on a product family design methodology.^6^ Gonzalez-Zugasti and Otto^22^ describe a product family approach to NASA’s exploratory space missions using a two-stage optimization-based approach.^22^ Simpson^23^ employed a genetic algorithm to perform a product family design approach to airplane selection.^23^ Pirmoradi et al.^24^ used a black-box optimization approach to craft a variety of universal electric motor designs.

Reported approaches for optimizing product family design have largely been heuristic and, despite the documentation of significant success in other industrial manufacturing settings, have not been widely applied to chemical process design; our work has focused largely on these two knowledge gaps. Stinchfield et al.^7^ described the first extensive, optimization-based approach to process design using process family design (an adoption of product family design to process systems engineering). The discretization approach was modeled using an MILP to design a family of refrigeration systems;^8^ this was extended by Stinchfield et al.,^7^ demonstrating the simultaneous design of 76 water desalination systems and 63 carbon capture systems. Stinchfield et al.^10^ formalized the MINLP that represents the general optimization-based approach to the process family design problem. The MINLP representation of the process family design problem is challenging to solve directly; recent work has focused on decomposition-based approaches, as documented by Stinchfield et al.,^25^ to solve larger problem instances. In the paper by Stinchfield et al.,^10^ the authors first proposed modifications to convert the MINLP to an MILP by embedding ReLU surrogates to design a family of refrigeration systems. In this work, we formalize this approach and embed a variety of machine learning surrogates in the design families of water desalination systems and carbon capture systems.

The literature for embedding machine learning models as surrogates in both simulation and optimization is extensive. Researchers utilize mixed-integer linear programming and nonlinear programming formulations for neural networks,^26−30^ tree ensembles,^31−34^ and decision trees.^9,35,36^ Additionally, several tools such as the Optimization & Machine Learning Toolkit (OMLT)^3^ automatically embed trained machine learning models within algebraic modeling languages such as Pyomo.^4^ These tools allow for streamlined adoption of these techniques across several applications in operations research and engineering.

Applications utilizing machine learning models are emerging in all areas of engineering. Patel et al.^37^ present the Neur2SP framework that utilizes trained neural networks as approximations for the value function in a two-stage stochastic program. They demonstrate this framework on capacitated facility location problems. Bertsimas et al.^38^ embed regularized linear regression models, random forests, and support vector machines to design chemotherapy regimens. Wu and Maravelias^36^ utilize linear model decision trees for maintenance optimization of building cooling systems. Zhang et al.^39^ embed graph neural networks for computer-aided molecular design. These contributions are a small sample of the existing literature utilizing machine learning models within optimization problems. In our work, we demonstrate this methodology and utilize OMLT in process family design to reduce the upfront computational burden.

## Process Family Design

In this section, we describe process family design and the types of problems toward which this methodology is targeted. We supplement the problem description with a small illustrative case study and introduce some of the vocabulary, sets, and variables used to define and solve the process family design problem.

### Problem Description

Process family design can be used when there is a need to deploy multiple instances of a particular *process system architecture*. The process system architecture captures the block flow diagram describing a particular process system; in other words, it details all of the general *unit module types* and how they connect within the system. A unit module type refers to one general unit operation within the process system architecture, while a *unit module design* describes all specifications required to manufacture an instance of a unit module type. For example, the unit module type could be a compressor, while the unit module design would be the specific design flow, material, and size of the compressor to be manufactured.

A process family, , is a set of process variants that span the set of design criteria required by all of the potential installations. Each *process variant* is defined by a set of design requirements. The design requirements refer to specifications, such as environmental factors or customer demands, that the *process variant design* must be able to accommodate. Similar to the distinction between the unit module type and unit module design, the process variant describes the general design requirements, while the process variant design describes the exact specifications of the process system architecture to be manufactured.

Given a set of process variants *V* and a process system architecture, the goal is to determine the *process family design*; this defines the complete set of process variant designs for each of the variants *v* ∈ *V*. A key element of this method involves determining and using a set of common designs stored in the process platform  to generate cost savings due to standardization at the manufacturing level. By breaking down the process system architecture into a set of unit module types *M*, we can separate this set into two disjoint sets, *U* and *C* (where *U* ∩ *C* = ⌀ and *U* ∪ *C* = *M*). The set *C* is all *common unit module types*, where the designs of these unit modules for each variant must be selected from the pool of common designs in the platform, . The remaining set *U* is all of the *unique unit module types* that can be designed uniquely for a particular variant. The designation of a unit module type, *m* ∈ *M*, as common or unique must be determined a priori. Many valid methodologies can be used to reason between which label to assign to each unit; this is handled on a case-by-case basis.

To determine a valid process family, , the following criteria must be satisfied.

1. For each of the common unit module types, *c* ∈ *C*, the process platform, , has a number of fully specified designs available.
2. For each variant, *v* ∈ *V*, each common unit module type, *c* ∈ *C*, has selected exactly one unit module design from the platform, *P*.
3. For each variant, *v* ∈ *V*, each unique unit module, *u* ∈ *U*, has one fully specified unit module design.
4. For each variant, *v* ∈ *V*, all unit module types, *M*, have designs that form a process system satisfying variant *v*’s design requirements.

To construct a cost-optimal process family, we aim to determine which designs should be included within the process platform  along with the unit module designs *m* ∈ *M* for all of the variants *v* ∈ *V* such that the total cost of designing, deploying, and operating the process family  is minimized.

### Illustrative Problem Approach

**Figure 1:**
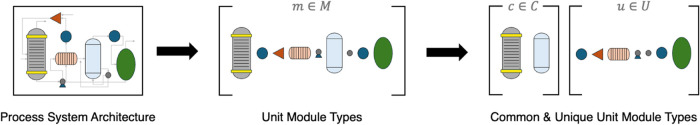
Process System Architecture broken down into Common and Unique Unit Module Types.

Consider a company that has six different locations for carbon-emitting chemical plants: Houston, Seattle, Boston, San Diego, Tampa, and Atlanta. This company intends to fit each plant with a carbon capture system. In Figure 1, consider the general carbon capture system broken down into the key unit module types. We elect to designate the absorber and the regenerator as part of the set of common unit module types *C* = [abs, regen], while those remaining will be unique unit module types, *U* = [mixer, cooler, pump, cond, heatex, flash].

**Figure 2:**
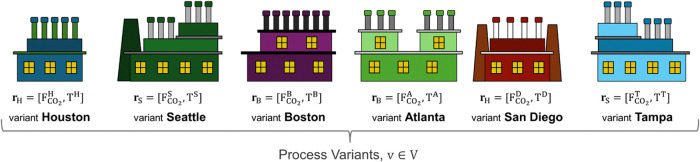
Set of Process Variants and Design Requirements.

As shown in Figure 2, we need to design a process family  with six variants. While the process system architecture remains the same, each of the six plants has a different set of design requirements. The composition of the flue gas is the same in all locations. This leaves two unique design requirements for each of the six variants *v* ∈ *V* = [H, S, B, A, D, T]; the first is the flow rate of the CO_2_-rich flue gas (*F*_CO_2__^*v*^) and the second is the outdoor design temperature where each plant is located (*T*^*v*^).

**Figure 3:**
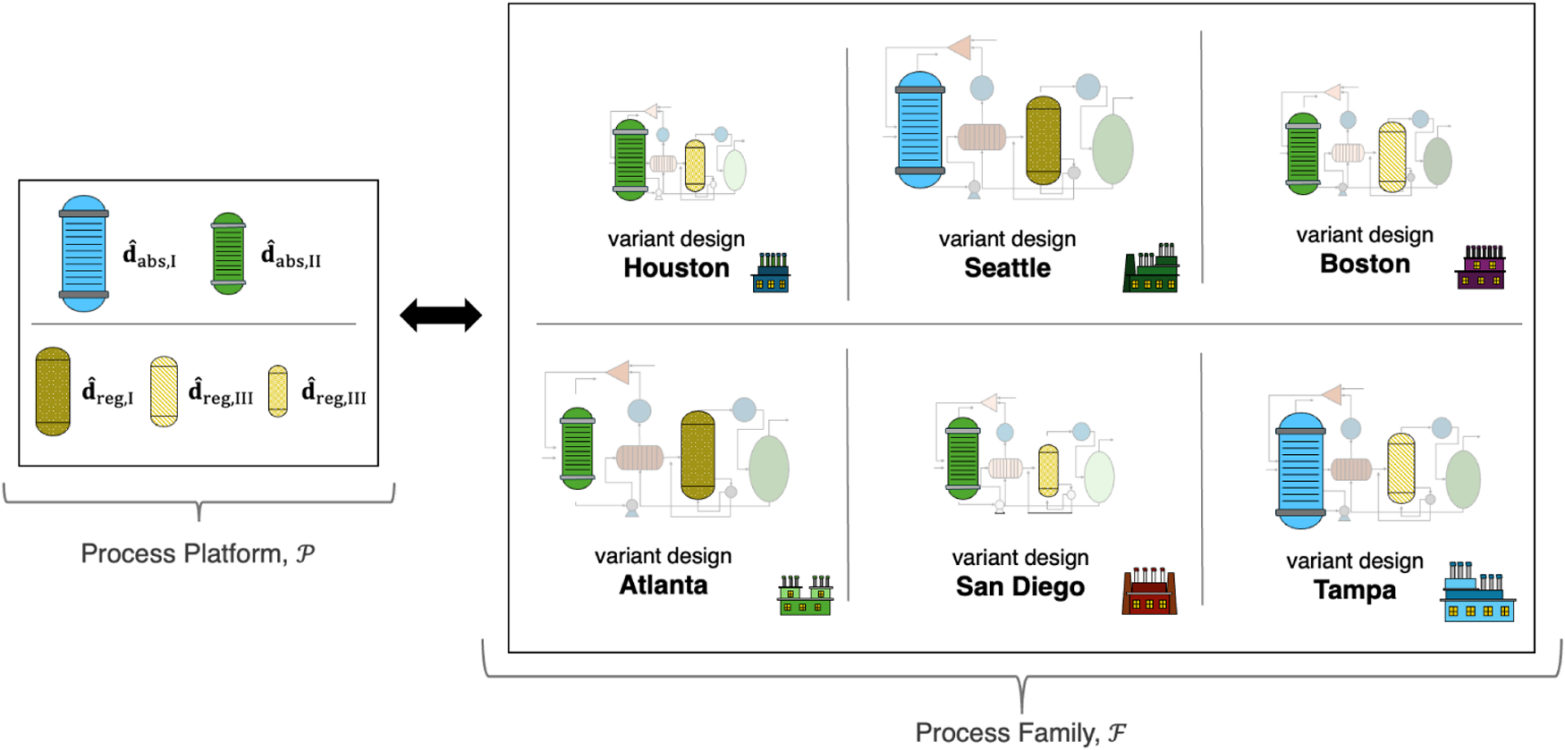
Process Platform  and Process Family .

To design the process family , we must determine the common designs in the platform ; simultaneously, we must assign the designs in the platform  that are used for each of the common unit module types *c* ∈ *C* = [abs, regen] at each variant *v* ∈ *V* = [H, S, B, A, D, T]. Consider the process platform to be composed of two absorber designs (specified through the vectors **d̂**_abs,I_ and **d̂**_abs,II_) and three regenerator designs (specified through the vectors **d̂**_reg,I_, **d̂**_reg,II_, and **d̂**_reg,III_), shown graphically on the left side of Figure 3.

Based on this process platform , we assigned the common unit modules *c* ∈ *C* for each variant *v* ∈ *V* as shown graphically on the right side of Figure 3. All of the remaining unique unit module types *u* ∈ *U* have designs that are fully specified for each variant *v* ∈ *V*.

For this small illustrative case study, the process family design introduced standardization when manufacturing some of the unit modules. Rather than designing six different absorbers, we only need to design two, producing design I twice and design II 4 times; a similar result occurs for the regenerator. Economies of numbers play a major part in reducing costs whenever something is produced more than once. Additionally, rather than having to design and manufacture six separate carbon capture systems, this chemical company was able to design all of them at once. For large chemical companies aiming to bring down costs and reduce timelines for deploying carbon capture devices across their suite of existing chemical plants, process family design can prove to be a pivotal manufacturing and design technique. For more realistic case studies, the number of variants comprising the family or the size of the platforms will be scaled up in comparison with the illustrative example presented here. For example, for a similar process family design breakdown as described for this illustrative example, our first case study is to design a family of 63 carbon capture facilities with 3 common regenerators and 3 common absorbers.

## Problem Formulation

In this section, we describe the computational- and optimization-based approach used to solve the process family design problem. We present the original MINLP formulation to represent the process family design problem^7^ We outline how we extended this methodology using ML surrogates and introduce our alterations to this formulation for embedding these ML surrogates in place of design equations.

### Nonlinear Generalized Disjunctive Formulation

The goal is to optimize the design of the process family  such that it is cost-optimal to deploy all variants *V*. The formulation, first proposed by Stinchfield et al.,^10^ can be described by the following MINLP
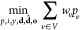
1a
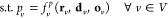
1b
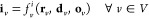
1c
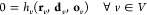
1d
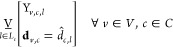
1e
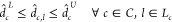
1f
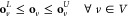
1g
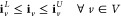
1h
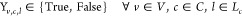
1i

We capture the annualized total cost of a variant *v* ∈ *V* in variable *p*_*v*_. Additionally, we parametrize each variant *v* ∈ *V* with an expected sales weight *w*_*v*_ to reflect the expected number of times a variant *v* ∈ *V* will be deployed. The stacked vector **d**_*v*_ contains a vector of design variables **d**_*v*,*m*_ for each unit module type *m* ∈ *M*. In this way, **d**_*v*_ contains all designs specific to variant *v* ∈ *V*, including those that are designed commonly (i.e., as part of the platform, **d**_*v*,*c*_ for all *c* ∈ *C*) and uniquely for each variant (i.e., **d**_*v*,*u*_ for all *u* ∈ *U*). In other words, **d**_*v*_ is the concatenation of all common design vectors with all unique design vectors for a particular variant *v* ∈ *V* (i.e.,**d**_*v*_ = (**d**_*v*,*c*_, **d**_*v*,*u*_)). We optimize the weighted costs of all variants by determining the standardized designs in the platform (**d̂**_*c*,*l*_), the unit module designs for all modules *m* ∈ *M* at all variants *v* ∈ *V* (**d**_*v*_), and each variant’s operating conditions (**o**_*v*_).

Common unit module designs are selected from the platform  via a binary decision variable, Y_*v*,*c*, *l*_. This variable determines if the common unit module type *c* ∈ *C* at variant *v* ∈ *V* should have design **d̂**_*c*,*l*_. If this is True, then the design vector is **d**_*v*,*c*_ = **d̂**_*c*,*l*_. We must select exactly one design for each variant *v* ∈ *V* and each common unit module type *c* ∈ *C*. Furthermore, we must consider the fact that not all designs or combinations of designs for the common unit module types offered within the platform will be able to satisfy all requirements **r**_*v*_ for a particular variant *v* ∈ *V*. To ensure that such an infeasible combination of designs for the common unit module types *C* are not selected for variant *v* ∈ *V*, we introduce the performance indicator ***i***_*v*_. This variable, coupled with constraint (1h), ensures that the variants meet the required performance specifications.

The objective (1a) minimizes the weighted total annualized cost of all variants, *v* ∈ *V*. For each variant *v* ∈ *V*, (1b) defines the total annualized cost equations, (1c) calculates the performance indicator, and (1d) is the system of equations defining the process system. Constraints (1b), (1c), and (1d) are functions of the design requirements **r**_*v*_, unit module design variables for all module types *M* (**d**_*v*_), and operating variables (**o**_*v*_). Constraint (1e) decides, for common unit module type *c* at variant *v*, which design **d̂**_*c*,*l*_ offered in the platform  will be selected. For a particular common unit module type *c*, the disjunct associated with label *l* that is selected (i.e., *Y*_*v*,*c*,*l*_ = True) equates design vector **d**_*v*,*c*_ for variant *v* to the design vector corresponding to the common unit module design selected **d̂**_*c*,*l*_. The common unit module designs **d̂**_*c*,*l*_ offered in the platform  are continuous variables; (1f) constrains these variables to specified boundaries for each common unit module type *c*. Similarly, the performance indicators **i**_*v*_ and operating variables **o**_*v*_ for all variants *v* ∈ *V* are constrained to feasible limits by (1g) and (1h).

### Embedded Machine Learning Surrogates Problem Formulation

The formulation presented in (1) is an MINLP. Nonlinear functions originate largely from the equations describing cost relationships (1b), determination of performance indicators (1c), and the functions that define the physics of the process system (1d). Optimizing the system of equations that represent a process system (i.e., eqs (1b–1d)) is challenging. Even for relatively small systems, there are a large number of constraints coupled with complex nonlinearities. When we consider directly trying to solve Formulation (1) with this set of equations included directly within the optimization formulation, it can quickly become intractable without more advanced decomposition or tailored algorithmic approaches.

In this formulation, we propose optimizing multiple instances of a process system model and solving them simultaneously. This formulation has the potential to become computationally intractable; in addition, (1b–1d) often introduce nonconvex elements, which adds additional challenges to an already difficult formulation. Rather than working on methods that include explicit nonlinear equations that introduce these complexities, we elect to replace them with well-trained piecewise linear ML surrogates.

We propose sampling the continuous design space of each of the process variants. We do this by treating the common unit module designs **d̂**_*c*,*l*_ along with the variant requirements **r**_*v*_ as inputs to the surrogates. Since we require two different surrogates, the prediction will be either the classification of feasibility for this design combination at variant *v* ∈ *V* or the predicted annualized total cost of this design for a particular variant *v* ∈ *V*. Given a candidate set of common unit module designs and variant *v* ∈ *V* requirements **r**_*v*_, we first generate a data set of the ground truth for feasibility and the annualized total cost by fixing these inputs within the explicit nonlinear system model i.e., (1b–1d) and solving for the unique designs **d**_*u*_, operating variables **o**_*v*_, feasibility indicators **i**_*v*_, and the annualized total cost *p*_*v*_. Our surrogates aim to predict **i**_*v*_ and *p*_*v*_ given **r**_*v*_ and a proposed **d̂**_*c*,*l*_.

We chose to use ML surrogates instead of, for example, black-box models because ML surrogates can be reformulated explicitly as math programs. Given the set of trained parameters (θ) for a particular ML surrogate, we can take the exact functional form of the surrogate parametrized with θ and embed it directly within an optimization formulation. By employing *piecewise linear* machine learning surrogates (such as ReLU-activated neural networks, linear model decision trees, and gradient-boosted decision trees), we can formulate the ML surrogate as an MILP. Piecewise linear surrogates have shown documented success in capturing (with good accuracy) complex, nonlinear process system dynamics.^9^ By eliminating nonlinear eqs 1b–1d with an MILP representable ML surrogate approximating them, we now aim to solve an MILP rather than an MINLP.

The following formulation shows the modified formulation that embeds ML surrogates, presented in (2)
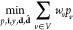
2a
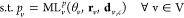
2b
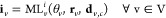
2c
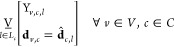
2d
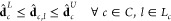
2e
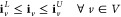
2f
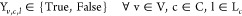
2g

In this formulation, the objective (2a), disjunctive decisions (2d), platform design ranges (2e), and performance indicator ranges (2f) are identical to constraints found in formulation (1). We replaced the nonlinear eqs 1b and 1d with ML surrogates, as shown in (2b) and (2c). These two systems of equations exactly represent the *trained* piecewise linear machine learning models (ML_*v*_^*p*^ and ML_*v*_^*i*^) that predict the cost and performance indicators for each variant *v* ∈ *V*. We automatically generate this transformation using the open-source package OMLT to reformulate given trained ML surrogates (i.e., trainable parameters are fixed) into different math programming formulations that can easily be embedded within an overall optimization problem modeled in Pyomo.^3,4^ The inputs to the surrogates are the common unit module designs **d**_*v*,*c*_ selected for a variant *v*, the parametrized design requirements **r**_*v*_, and the trained ML surrogate parameters for variant *v*.

In this approach, ML surrogates are used to predict the feasibility and costs for a given set of variant requirements as shown in (2c). Care should be taken to ensure that the surrogates are sufficiently accurate, and after the optimization is complete, the solution should be validated using the rigorous model. As with any surrogate-based approach, if the model is insufficiently accurate, it may be necessary to improve the surrogate model (e.g., with additional data or training) or consider a higher threshold on the feasibility indicators, as shown in (2f).

Within the IDAES organization^40^ on GitHub, we have developed a Python package called process family under the repository for multi-variant optimization (IDAES-mvo).^41^ This package can automatically generate the discretized formulation^7^ and the ML surrogate formulation presented in eq (2) using Pyomo^4^ for a given data set describing a process family design problem. We utilize OMLT^3^ for the automatic transformation of ML models into their MILP representations and Pyomo.GDP^42^ for automatic big-M reformulation of disjunctions in the ML surrogates formulation.

## Case Studies

In this section, we present two case studies to demonstrate the approaches detailed in previous sections. For each case study, we present a general process system description, outline the process system architecture, and discuss the details of the process family design. Additionally, we present the data collection, training, and hyperparameter tuning for each machine learning surrogate. The two case studies demonstrated are an MEA solvent-based carbon capture system and a water desalination system intended for the produced water.

### Carbon Capture Case Study

In our first case study, we use an MEA-based carbon capture system, modeled in Aspen, and design 64 variants given a variety of CO_2_ concentrations in the flue gas and different flue gas flow rates.

#### Process System Architecture

This work adapts a process model,^43^ developed by the U.S. Department of Energy’s Carbon Capture Simulation Initiative (CCSI),^44^ of the aqueous MEA solvent-based CO_2_ capture system. In previous works,^45,46^ this model was validated at the scale of 0.5 MWe with process data collected at the National Carbon Capture Center (NCCC) representing a wide range of operating conditions and variable absorber configurations.

In this process, the CO_2_-rich flue gas from the point source enters the bottom of the absorber tower where it is contacted countercurrently with the solvent system, or 30 wt % aqueous MEA in this case. The CO_2_-lean solvent enters the top of the absorber, flows downward, and captures CO_2_ from the upward-flowing gas through reactive absorption. The absorber is operated in a regime in which transfer of CO_2_ from the gas to liquid is thermodynamically favorable, generally with atmospheric pressures and temperatures of 30–45 °C. Since the reaction between CO_2_ and the amine solvent is exothermic, a temperature bulge occurs in the column with temperatures reaching as high as 70 °C, which reduces the driving force for CO_2_ absorption and can result in a pinch condition. Although not shown in this schematic, the process model includes the option of using solvent intercoolers to reduce the temperature bulge by withdrawing a portion of the solvent, cooling it in an external heat exchanger, and returning it to the column. The clean flue gas, with a reduced CO_2_ concentration, exited the top of the absorber column.

The CO_2_-rich solvent exiting the bottom of the absorber is pumped to a higher pressure and heated to a higher temperature (typically 100–110 °C) to obtain conditions thermodynamically favorable for stripping CO_2_ from the solvent. The heating is accomplished by heat exchange from the CO_2_-lean solvent exiting the bottom of the stripper to the CO_2_-rich solvent entering the top of the stripper. The hot CO_2_-rich solvent enters the top of the stripper, where the endothermic reaction in which CO_2_ is separated from the solvent occurs and steam supplied to the reboiler at the bottom of the column provides the energy input. The CO_2_/H_2_ O mixture exiting the top of the stripper is condensed, resulting in a nearly pure CO_2_ stream (generally 98%) that is sent for storage. The liquid reflux is returned to the top of the stripper column. The CO_2_-lean solvent exiting the bottom of the stripper enters the lean-rich heat exchanger, where it is cooled by the CO_2_-rich solvent. An additional cooler is generally required to reduce the temperature to absorber conditions. After the addition of makeup water and solvent to compensate for losses in the process, the CO_2_-lean solvent is returned to the top of the absorber column.

#### Process Family Design

**Figure 4:**
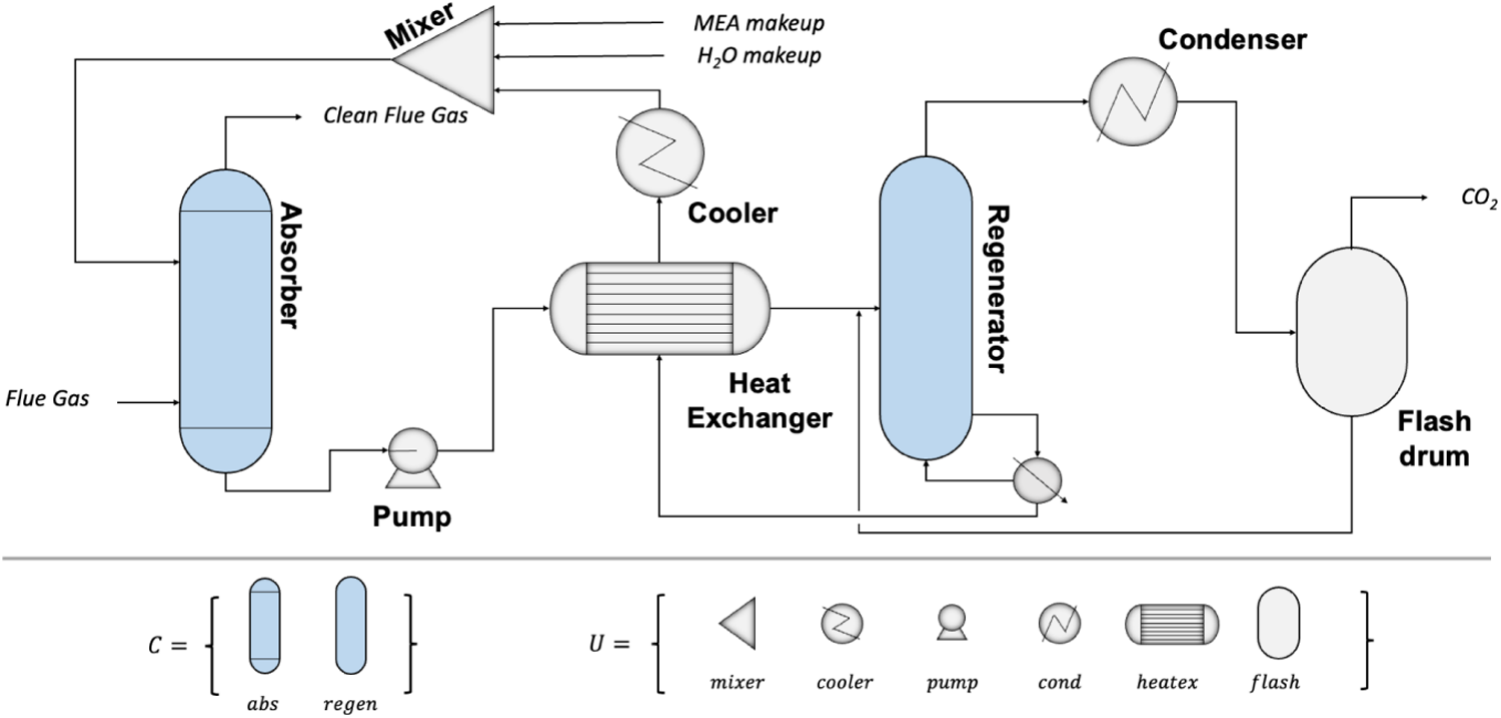
Carbon Capture Process System Architecture.

The process model described above is represented schematically in Figure 4. The process system architecture must be broken down into the set of unit module types, *M*, and then partitioned into the two sets of unique *U* and common *C* unit module types. The set of unit module types for the carbon capture system is represented as M = [abs, mixer, cooler, pump, heatex, regen, cond, flash]. For this case study, we selected the absorber (abs) and the regenerator (regen) to be designed commonly as a part of the process platform ; the set of common unit module designs is, therefore, *C* = [abs, regen]. The remaining unit module types make up the unique unit module types *U* = [mixer, cooler, pump, cond, heatex, flash].

Recall that the process variant *v* ∈ *V* is defined by a set of design requirements **r**_*v*_ that the process variant design must be able to satisfy. In this case study regarding carbon capture, (1) the concentration of CO_2_, denoted by *c*_CO_2__, and (2) the flow rate of the CO_2_-rich flue gas, denoted by *f*_CO_2__, significantly impact many elements of the process design. These two variables will be the design requirements (i.e., **r**_*v*_ = (*c*_CO_2__, *f*_CO_2__)) differentiating each process variant *v* ∈ *V* in the process family . Each of the 63 process variants *v* ∈ *V* (where |*V*| = 63) will be defined by a unique set of the CO_2_ concentration (*c*_CO_2__) and flue gas flow rate (*f*_CO_2__). We selected 7 different flue gas flow rates, from 2000 kg/h to 3200 kg/h, based on the typical flow rates expected at a pilot plant-scale carbon capture facility. In addition, we considered 9 different CO_2_ concentrations, from 5 to 29%, incremented by 3% for 9 different percentages. The range was selected based on the average CO_2_ concentrations that can be expected in industry from a variety of applications (cement plants, NGCC, etc.). Taking the cross product of the 7 different flow rates and 9 different CO_2_ concentrations, we define the set of 63 process variants.

#### Sampling of the Design Ranges

For the discretization formulation presented by Stinchfield et al.,^7^ we developed a set of feasible design alternatives *A*_*v*_ by running simulations of this model in Aspen Plus. This particular approach to solving the process family design problem relied on a defined set of *candidate designs S*_*c*_ for each of the common unit module types *c* ∈ *C*. From these sets of candidate designs, we then constructed the set of *all possible combinations* of candidate unit module designs *Q*; in other words, the set *Q* comprises the Cartesian product of each *S*_*c*_ for all *c* ∈ *C*. For each potential combination *q*∈*Q* at each variant *v* ∈ *V*, a simulation of the Aspen model was performed (where the common designs as defined by *q* ∈ *Q* and the design requirements *r*_*v*_ were fixed) to determine the operating variables *o*_*v*_, unique designs, feasibility indicators *i*_*v*_, and total annualized cost *p*_*v*_. The set of alternatives *A*_*v*_ is the subset of all possible combinations as defined by *Q* that are feasible for a particular variant *v* ∈ *V* (i.e., *A*_*v*_ ⊆*Q*). While the sets *Q* and *A*_*v*_ originally functioned as a parametrization of the optimization problem, for this approach, we used this data set as the training data for the surrogate models to predict ***i***_*v*_ and ***p***_*v*_.

We must investigate all *possible* combinations of candidate designs *Q* for the common unit modules in set *C*. For the absorber and regenerator, we fix the *L*/*D* ratio based on pilot plant parameters at NCCC^45,46^ and consider the column diameter as the design variable. While we will label each design by the value of the column diameter design variable, it is important to note that because the *L*/*D* ratio is fixed, the height also changes for each column. In any particular process family design, the number of elements in each common unit design is up to the discretion of the engineer.

We consider six diameters for the absorber (*S*_absorber_ = [0.5, 0.6, 0.7, 0.8, 0.9, 1.0 m]) and eight diameters for the regenerator (*S*_regenerator_ = [0.3, 0.4, 0.5, 0.6, 0.7, 0.8, 0.9, 1.0 m]) as the sets of candidate unit module designs. For each variant, we perform one simulation for each possible combination *q* ∈ *Q* of the absorber diameters and regenerator diameters considered (*Q* = *S*_absorber_ × *S*_regenerator_ = [(0.5, 0.3 m), ···, (1.0, 1.0 m) ]). In total, this is 6 × 8 = 48 simulations *per variant*. Given that we are considering 63 variants in total, this requires 6 × 8 × 63 = 3024 simulations.

Ideally, this model would have been optimized over the CO_2_ lean loading. However, given the simulation framework, we elected to consider five CO_2_ lean loading concentrations (0.16, 0.17, 0.18, 0.19, 0.20) for each candidate design combination *q* ∈ *Q* (i.e., we now perform 3,024 × 5 = 15,120 simulations in total). Once all simulations were performed, we selected one CO_2_ lean loading for each valid alternative *a* ∈ *A*_*v*_ that yielded the lowest total cost (this is captured in variant-specific operating variable **o**_*v*_).

Given the 63 different process variants we wish to design, six absorber designs, eight regenerator designs, and five CO_2_ lean loadings considered, this case study required 15,120 simulations. The total annualized cost was computed using correlations within the IDAES framework.^40^ Obtaining this data set took approximately 250 h with homotopy-type methods to achieve good convergence. This data set was used to fit surrogates for Formulation (2).

#### Machine Learning Surrogates

As described earlier, we must embed two surrogates: One performing a classification task ML_*v*_^*i*^ and another performing a regression task ML_*v*_^*p*^. The classification surrogate will predict the feasibility indicator ***i***_*v*_, which determines if a set of common unit module designs (one for each *c* ∈ *C*) is a *feasible* combination of units at a particular process variant *v* ∈ *V*. In other words, given a set of common designs **d**_*v*,*c*_ for the subset of unit module types that are designated to be in the common set *C*, there are unique unit module designs and operating conditions that permit an overall process design that is operational at the design requirements ***r***_*v*_ specified by the variant *v* ∈ *V* at hand? If we find that the answer to the classification prediction is True (i.e., it is feasible), then we need a regression surrogate to estimate what the total annualized cost *p*_*v*_ of designing and operating that particular variant *v* ∈ *V* (again with the common unit module designs **d**_*v*,*c*_ and design requirements ***r***_*v*_ as input parameters) would be.

For the classification surrogate ML_*v*_^*i*^ (θ, **r**_*v*_, **d**_*v*,*c*_), we trained the parameters θ and embedded the final neural network (NN) with a single layer of 15 ReLU-activated nodes and a single logistic output node using Keras.^1^ Once training was completed, the logistic output activation was removed from the automatic reformulation performed by OMLT^3^ and replaced with a linear activation. This step was taken because we aimed to use a piecewise linear surrogate within the optimization framework.

For the cost predicting surrogate ML_*v*_^*p*^ (θ, **r**_*v*_, and **d**_*v*,*c*_), we chose a Gradient-Boosted Decision Tree (GBDT). We built and trained the GBDTs using LightGBM.^47^ Hyperparameters θ of the GBDTs were found by using a grid search. For a GBDT, trainable hyperparameters are maximum depth, minimum data used per leaf, and total number of trees in the ensemble. The hyperparameters selected for both case studies, along with their training and testing accuracies, for the GBDTs are shown in Table 1.

**Table 1:** Regression GBDT Statistics for Carbon Capture

depth	samples	trees	train MSE	test MSE
10	1	100	2.89 × 10^–5^	1.04 × 10^–4^

### Water Desalination Case Study

In the second case study, we used a water desalination process system and designed 76 variants; each variant consists of a unique combination of a specified salt concentration and a flow rate describing the inlet brine. We describe the process system architecture built in Pyomo,^4^ followed by the general setup used to design the process family and how the machine learning surrogates were trained.

#### Process System Architecture

A promising technology for high-salinity produced water is evaporation and mechanical vapor recompression.^48^ For this case study, we use a mechanical vapor recompression model implemented in Pyomo^4^ as part of the PARETO framework.^49^ We only consider a treatment unit that consists of one effect evaporator and a single-stage adiabatic compressor.^48^

The inlet of the unit is assumed to be produced water, which contains many different dissolved solids. This inlet stream first enters the shell side of the evaporator to be split into one of two streams: a concentrated brine liquid and a vapor stream. The vapor from the evaporator goes on to be compressed further by using an adiabatic compressor that ultimately converts it into superheated steam. We included a minimum recycle line, which ensures that we can operate at different specified design flow rates. The superheated steam is injected into the tubes of the evaporator to be condensed; this provides the heat needed for evaporating the produced water fed into the shell side of the evaporator. The condensate is removed from the system as a freshwater stream as all dissolved solids have been removed. Operating costs of the system were determined using the cost of electricity due to compressor operation, while equations for estimating capital costs were obtained using the IDAES Integrated Platform.^40^

#### Process Family Design

**Figure 5:**
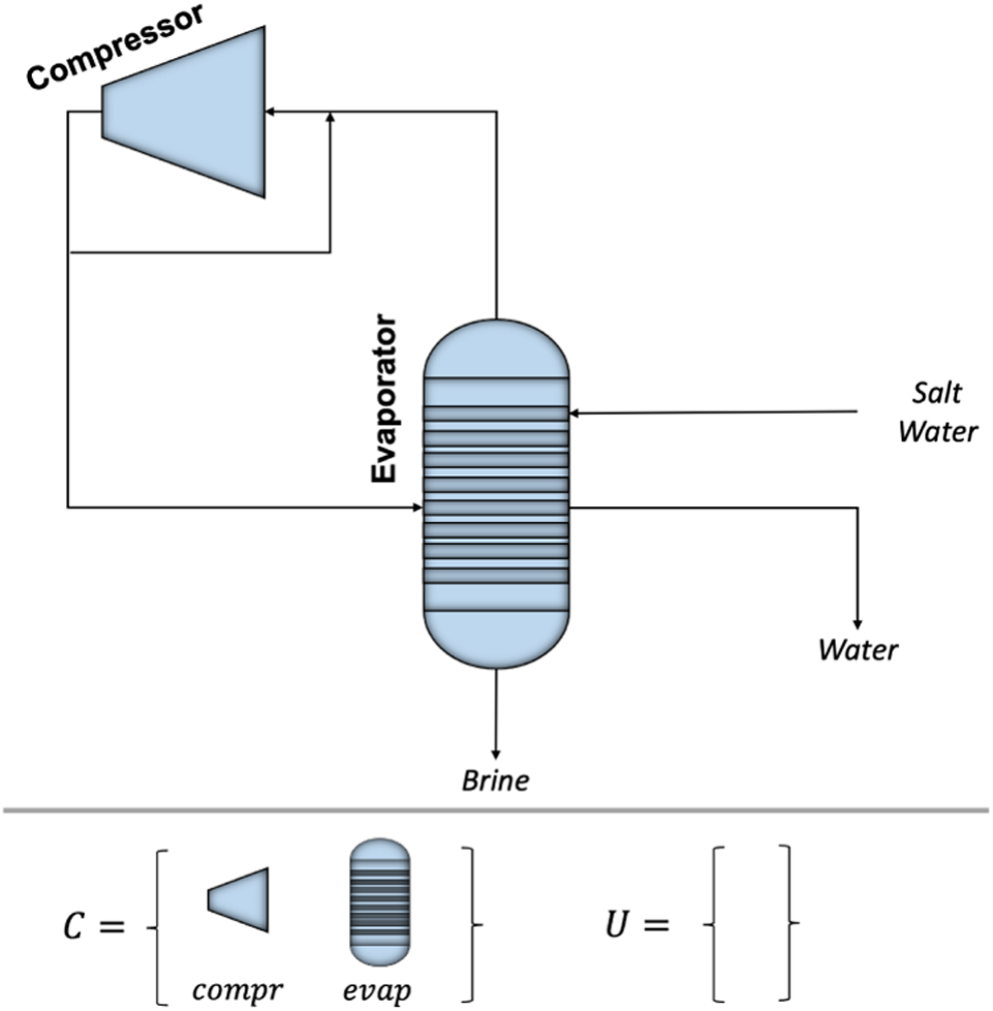
Water Desalination Process System Architecture.

The water desalination process is schematically represented in Figure 5.

For performing the process family design approach, from the set of unit module types M = [evap, compr], we define the set of common unit module types *C*. In this case study, both the evaporator and the compressor will be designed as common, and none will be designed to be unique (i.e., *C* = M = [evap, compr] and *U* = ⌀).

We aim to design 76 different water desalination units. Each variant *v* ∈ *V* has unique design requirements **r**_*v*_ defined by a feed flow rate *f*_*v*_ and a feed salt concentration *c*_*v*_ (i.e., **r**_*v*_ = (*f*_*v*_, *c*_*v*_)). To define a variant, we considered a set of 76 randomly sampled salt concentrations between 20 and 150 g/kg, which represents typical salinity ranges acceptable for this system design and additionally aligns with samples of produced water.^49^ We considered 76 randomly sampled flow rates between 0.1 and 1 kg/s (i.e., 55 bbls/day and 500 ggls/day). We coupled these two sets of 76 random samples to develop the set of 76 variants *V*.

#### Sampling of the Design Ranges

We aim to design both the compressor and the evaporator commonly as part of a process family design scheme. As described in detail for the previous case study, we must define a set of candidate designs *S*_*c*_ for each of the common unit module types *c* ∈ *C* = [evap, compr] considered. For the evaporator, we considered 20 different candidate evaporator areas (ranging uniformly from 20 to 400 m^2^). For the compressor, we considered 20 different candidate design flows (ranging uniformly from 0.05 to 1 kg/s). The set of possible candidate design combinations *Q* is, therefore, the Cartesian product of these 20 candidate evaporators *S*_evap_ and the 20 candidate compressors *S*_compr_, yielding 400 candidate design combinations (|*Q*| = 400). Given that we aim to design 76 variants, this requires us to solve an optimization |*V*| × |*Q*| = 30,400 times. Each instance was modeled in Pyomo v6.5.0^4^ and solved using IPOPT v3.13.2.^50^ Each subsequent optimization instance was initialized from the solution of the previous point.

#### Machine Learning Surrogates

For the classification surrogate ML_*v*_^*i*^ (θ, **r**_*v*_, **d**_*v*,*c*_), we selected a Linear Model Decision Tree (LMDT) for the water desalination case study. We built and trained the LMDT using the open-source Python package linear tree.^51^ We tuned the hyperparameters θ of the LMDTs using a grid search, assessing combinations of parameters for the maximum number of bins, maximum depth, and minimum number of samples per leaf. Selected hyperparameters and their training and testing accuracies for the LMDT used are shown in Table 2.

**Table 2:** Classification LMDT Statistics for Water Desalination

bins	depth	samples	train acc.	test acc.
100	20	25	99.19%	99.05%

For the cost predicting surrogate ML_*v*_^*p*^ (θ, **r**_*v*_, **d**_*v*,*c*_), we elected to use a Gradient-Boosted Decision Tree (GBDT). We built and trained the GBDTs using LightGBM.^47^ Hyperparameters θ of the GBDTs, as was the case with the LMDT, were found by using a grid search. For a GBDT, trainable hyperparameters are maximum depth, minimum data used per leaf, and total number of trees in the ensemble. The hyperparameters selected, along with their training and testing accuracies, for the GBDTs are shown in Table 3.

**Table 3:** Regression GBDT Statistics for Water Desalination

depth	samples	trees	train MSE	test MSE
10	1	100	2.74 × 10^–5^	4.38 × 10^–5^

## Results and Discussion

Here, we describe the results of the two case studies defined above. We describe the optimal process families , process platforms , and computational performances. Additionally, we discuss the results in the context of the discretization approach proposed and solved by Stinchfield et al.^7^ We modeled both case studies as Problem 2 in Pyomo v6.6.1^4^ and solved them using Gurobi v10.0.1^52^ on a Macbook Air(M1,2020) with an Apple M1 Core with 8 physical cores. Automatic big-M reformulation of the disjunctions in Problem (2) were handled by the Pyomo package extension, Pyomo.GDP.^42^ The Optimization & Machine Learning Toolkit (OMLT)^3^ provided automatic transformations of ML surrogates into MILP representations.

### Carbon Capture Results

For this carbon capture process family design problem, we limited the number of designs allowed for the common unit module types *C* = [abs, reg] to be 3 each; in other words, the process platform  could contain no more than 3 absorber designs and 3 regenerator designs and |*L*_*c*_|= 3 for all *c* ∈ *C* = [abs, reg]. We set the threshold for feasibility indicators **i**_*v*_ to be 0.5. The optimal common designs for the absorber were those with diameters of 0.5, 0.65, and 0.74 m; additionally, the optimal common regenerator designs were determined to have diameters of 0.3, 0.45, and 0.62 m. Recall that the surrogates predict continuous design values from the surrogated space; the values reported here were rounded to 2 decimal places.

The resulting optimization formulation had 28,734 continuous variables and 2,898 binary variables. There were 58,783 constraints in total. It took approximately 15 min to train the surrogate models and just 25 s to solve the optimization model.

**Figure 6:**
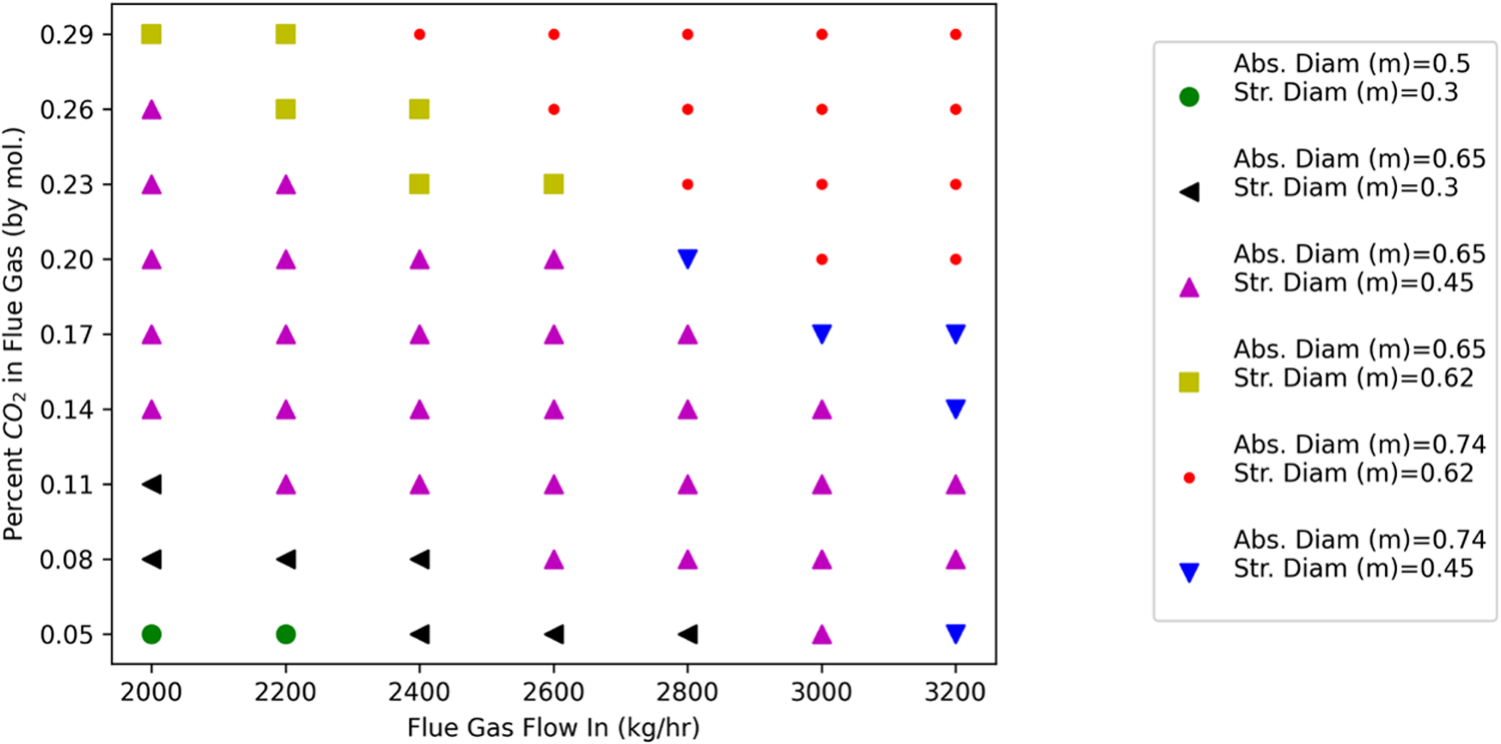
Carbon Capture Process Family.

Recall the process platform  was designed simultaneously with the process family . The optimal process family is shown in Figure 6.

The *x*-axis corresponds to a particular flue gas flow rate and the *y*-axis corresponds to a particular CO_2_ concentration in the flue gas. Each (*x*, *y*) point corresponds to exactly one variant *v* ∈ *V* of the 63 carbon capture processes considered. The shape and color of each point indicate which combination of platform designs were selected for the common unit modules of that particular variant *v* ∈ *V* and can be decoded by following the associated legend. Additionally, the unique unit module designs *U* = [mixer, cooler, pump, cond, heatex, flash] and operating conditions **o**_*v*_ are determined for each variant *v* ∈ *V*, although they are not shown in the figure.

Based on the optimal process family  shown in Figure 6, increasing the flue gas flow rate is correlated with an increase in the absorber size and some increases in the stripper size. Similarly, as the percentage of CO_2_ increases, there is a need for larger absorbers and strippers. The biggest factor for an increase in the stripper size is when both the flue gas flow rate and the percentage of CO_2_ are increased simultaneously. This follows the logical assumption that the size of the carbon capture plant must increase when the demand for removal (due to either processing more flue gas or having to remove higher amounts of CO_2_) is increased.

### Water Desalination Results

For this case study, we limited the number of designs allowed for the common unit module types *C* = [evap, compr] to 3 each (i.e.,|*L*_*c*_|= 3 for all *c* ∈ *C* = [evap, compr]). The optimal common designs for the evaporator areas were 51.63, 194.29, and 276.60 m^2^; additionally, the optimal common compressor designs were determined to have design flows of 0.25 0.53, and 0.86 kg/s. These results were rounded to 2 decimal places.

The resulting optimization formulation had 32,306 continuous variables and 10,868 binary variables. There were 128,596 constraints in total. It took approximately 20 min to train the surrogate models and just 215 s to solve the optimization model.

**Figure 7:**
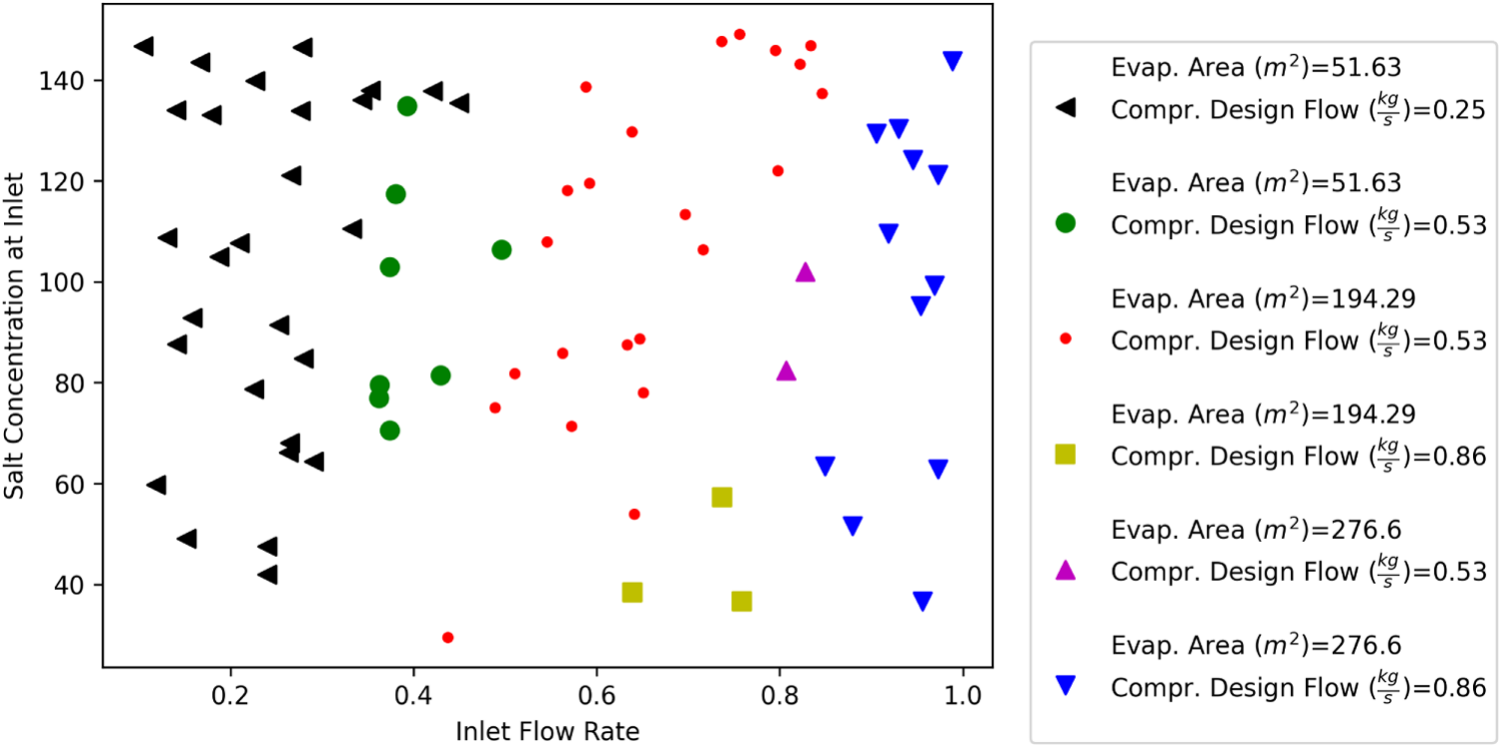
Water Desalination Process Family.

This optimal process platform  was designed simultaneously with the process family . The optimal process family is shown in Figure 7.

The *x*-axis corresponds to a particular brine inlet flow rate and the *y*-axis corresponds to a particular salt concentration at the inlet of the brine. Each (*x*, *y*) point corresponds to exactly one variant *v* ∈ *V* of the 76 water desalination processes considered. The shape and color of each point indicate which combination of platform designs were selected for the common unit modules of that particular variant *v* ∈ *V* and can be decoded by following the associated legend. Additionally, the operating conditions **o**_*v*_ are determined for each variant *v* ∈ *V*, although they are not shown in the figure.

Based on the optimal process family  shown in Figure 7, the relationship between inlet flow rate, salt concentration, evaporator area, and compressor design flow can be observed. Unlike the carbon capture case study, we did not uniformly select design requirements **d**_*v*_ for each variant *v* ∈ *V*, so the results have a nonuniform display of each variant’s common design decisions. Based on Figure 7, it is clear that the inlet flow rate design requirement has a significantly larger impact on the designs of both the evaporator area and compressor design flow when compared to changes in the salt concentration at the inlet. This makes logical sense as the evaporator area and compressor design flow must increase to handle higher flow rates.

## Conclusions

In this article, we present a novel approach for solving a process family design problem using embedded machine learning surrogates. We demonstrated the benefits and success of this approach in two case studies, but it should be noted that the methodology outlined in this paper is general and can be applied to a wide range of industrial processes. The first case study demonstrated the design of a family of 63 carbon capture systems with three absorber and three regenerator designs. The second case study involved designing a family of 76 water desalination systems with 3 evaporator and 3 compressor designs.

This approach relies on embedding trained machine learning models within the overall optimization formulation in place of complicated nonlinear equations describing the physics, costing, and performance of a process system. The quality of training data and the fine-tuning approach to these ML models will significantly affect the quality of the predicted solutions. Extensions from our current approach might involve sampling more data points and training a different model per variant *v* ∈ *V*, rather than training on all data and using the same model for each variant. Sensitivity analysis could be performed on these points along with different types of models and how they are trained, to determine how the optimal process family is changed.

The discretization approach detailed by Stinchfield et al.^7^ can only improve the quality of solutions by increasing the number of candidate designs in *S*_*c*_. However, each additional candidate design directly increases the set of candidate combinations in *Q* and increases the number of optimization problems that must be performed a priori; it also increases the number of decision variables necessary for the final optimization problem. Here, adding more data to the training for the ML surrogates will increase the quality of the models without increasing the size of Formulation 2. Additionally, an important element of ML surrogates is their ability to capture complex functions with a sample of data; while the discretization approach will need to continue discretizing the design range to achieve better solutions, the ML surrogates do not need to discretize the space as finely to achieve strong results. In this way, the ML approach can theoretically decrease the computational requirements as we discretize further.

As with any surrogate-based modeling and optimization approach, the quality of the optimization solution is dependent on the accuracy of the surrogate model. If the data sets used to train the models are insufficient (e.g., low volumes of reliable data) or error-prone (e.g., using inaccurate models or experimental data), the resulting model may itself be insufficiently accurate, and the optimization solution may not be feasible for the real system. The volume of data needed is determined on a case-by-case basis, for which many rules-of-thumb have been reported in the ML community. Data quality and volume directly affect the quality of the trained surrogates, which in turn directly affect the quality of solutions for the process family design approach. Therefore, as with any surrogate-based approach, we recommend that the optimization solution be validated using the rigorous process model. If errors are significant or the designs are infeasible, then the surrogate should be improved (e.g., through additional data or training). A possible extension to this work could be investigating optimization-under-uncertainty approaches to directly address model quality in the optimization formulation.

Additional extensions to this work are considering decomposition and algorithmic approaches to solving large-scale problem instances of the ML-based approach and the discretization-based approach. While ML surrogates have been demonstrated to accurately capture process performance and cost (even in the presence of nonconvexities), explicit representation of the equation-oriented process system model within the optimization would be ideal. In recent years, we have investigated decomposition-based approaches for direct solution of the MINLP formulation originally posed.

## Disclaimer

This project was funded by the United States Department of Energy, National Energy Technology Laboratory, in part through a site support contract. Neither the United States Government nor any agency thereof, nor any of their employees, nor the support contractor, nor any of their employees, makes any warranty, express or implied, or assumes any legal liability or responsibility for the accuracy, completeness, or usefulness of any information, apparatus, product, or process disclosed or represents that its use would not infringe privately owned rights. Reference herein to any specific commercial product, process, or service by trade name, trademark, manufacturer, or otherwise does not necessarily constitute or imply its endorsement, recommendation, or favoring by the United States Government or any agency thereof. The views and opinions expressed herein do not necessarily reflect those of the United States Government or any agency thereof.
